# Modulation of Outer Hair Cell Electromotility by Cochlear Supporting Cells and Gap Junctions

**DOI:** 10.1371/journal.pone.0007923

**Published:** 2009-11-20

**Authors:** Ning Yu, Hong-Bo Zhao

**Affiliations:** Department of Surgery – Otolaryngology, University of Kentucky Medical Center, Lexington, Kentucky, United States of America; Hotchkiss Brain Institute, University of Calgary, Canada

## Abstract

Outer hair cell (OHC) or prestin-based electromotility is an active cochlear amplifier in the mammalian inner ear that can increase hearing sensitivity and frequency selectivity. In situ, Deiters supporting cells are well-coupled by gap junctions and constrain OHCs standing on the basilar membrane. Here, we report that both electrical and mechanical stimulations in Deiters cells (DCs) can modulate OHC electromotility. There was no direct electrical conductance between the DCs and the OHCs. However, depolarization in DCs reduced OHC electromotility associated nonlinear capacitance (NLC) and distortion products. Increase in the turgor pressure of DCs also shifted OHC NLC to the negative voltage direction. Destruction of the cytoskeleton in DCs or dissociation of the mechanical-coupling between DCs and OHCs abolished these effects, indicating the modulation through the cytoskeleton activation and DC-OHC mechanical coupling rather than via electric field potentials. We also found that changes in gap junctional coupling between DCs induced large membrane potential and current changes in the DCs and shifted OHC NLC. Uncoupling of gap junctions between DCs shifted NLC to the negative direction. These data indicate that DCs not only provide a physical scaffold to support OHCs but also can directly modulate OHC electromotility through the DC-OHC mechanical coupling. Our findings reveal a new mechanism of cochlear supporting cells and gap junctional coupling to modulate OHC electromotility and eventually hearing sensitivity in the inner ear.

## Introduction

The mammalian cochlea has auditory sensory hair cells and supporting cells. The supporting cells provide a mechanical support to hair cells achieving hearing function. It has been observed that the cochlear supporting cells in vivo can also influence the movement of the organ of Corti and play an important role in the control of hearing sensitivity [1], [2]. However, the cellular mechanism underlying this control remains unclear.

Deiters cells (DCs) are the cochlear supporting cells and are well-coupled by gap junctions [3], [4]. In vivo, the DCs act as a scaffold supporting outer hair cells (OHCs) standing on the basilar membrane [5]. OHCs in the mammalian cochlea have electromotility [6], which can rapidly alter cell length to boost the vibration of the basilar membrane and increase hearing sensitivity and frequency selectivity [7], [8]. OHC electromotility is directly driven by membrane potential. OHC electromotility also has tension dependence, influenced by membrane tension [9]–[11]. In this study, the effect of DC activity and gap junctional coupling on OHC electromotility was examined. We found that DC activation and gap junctional coupling can directly modulate OHC electromotility through DC-OHC mechanical coupling. The data reveal a new mechanism of cochlear supporting cells and gap junctional coupling on the regulation of OHC electromotility and eventually control of hearing sensitivity in the mammalian cochlea.

## Materials and Methods

### Animal preparation and cochlear cell isolation

The cochlear cells were freshly isolated from adult guinea pigs (250–400 g) [12], [13]. Briefly, the guinea pig was anaesthetized with an overdose of pentobarbital (200 mg/kg, i.p.) and the temporal bones were removed after decapitation. The isolated otic capsule was dissected in a normal extracellular solution (NES) (130 NaCl, 5.37 KCl, 1.47 MgCl_2_, 2 CaCl_2_, 25 Dextrose, and 10 HEPES in mM; 300 mOsm and pH 7.2). After removal of the bone and stria vascularis, the sensory epithelium (organ of Corti) was picked away with a sharpened needle and further dissociated by trypsin (0.5 mg/ml) for 2–3 minutes with shaking. Then, the cochlear cells were transferred to a recording dish. All experimental procedures were performed at room temperature (23°C) and conducted in accordance with the policies of University of the Kentucky Animal Care & Use Committee.

### Patch-clamp recording and nonlinear capacitance measurement

The DC-OHC pair was selected and classical dual patch clamp recording for gap junctional coupling was performed under the whole-cell configuration by using Axopatch 700A (Axon, CA) with jClamp (Scisft, New Haven, CT) [12], [13]. The patch pipette was filled with an intracellular solution (140 KCl, 10 EGTA, 2 MgCl_2_, 10 HEPES in mM; 300 mOsm and pH 7.2) and had initial resistance of 2.5–3.5 MΩ in bath solution. In the OHC patch pipette, K^+^ was substituted with Cs^+^ to block the potassium current for nonlinear capacitance (NLC) recording. For gap junctional recording, one cell was stimulated by voltage steps and another cell was held at −40 mV to measure the transjunctional currents [14]. The OHC NLC was measured by a two-sinusoidal method [13], [15]. The signal was filtered by a 4-pole low-pass Bessel filter with a cut-off frequency of 10 kHz and digitized utilizing a Digidata 1322A (Axon, CA). The capacitance was calculated by admittance analysis of the current response.

### Data processing and presentation

Data analysis was performed with jClamp and MATLAB [12], [13]. The voltage-dependent NLC was fitted to the first derivative of a two-state Boltzmann function: 
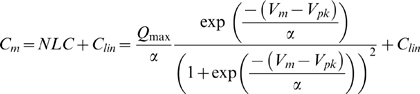
(1)where NLC is the nonlinear capacitance component associated with OHC electromotility, *Q_max_* is the maximum charge transferred, *V_pk_* is the potential that has an equal charge distribution and also corresponds to the peak of NLC, *α* is the slope of the voltage dependence. Membrane potential (*V_m_*) was corrected for electrode access resistance (*R_s_*). SigmaPlot software was used for figure plotting.

### Chemicals and chemical perfusion

All chemicals were purchased from Sigma Chemical Company (St. Louis, U.S.A.). A Y-tube perfusion system was used for applications of chemicals.

## Results

### Modification of OHC electromotility and frequency responses by electrical stimulations in Deiters cells

**Figure 1:**
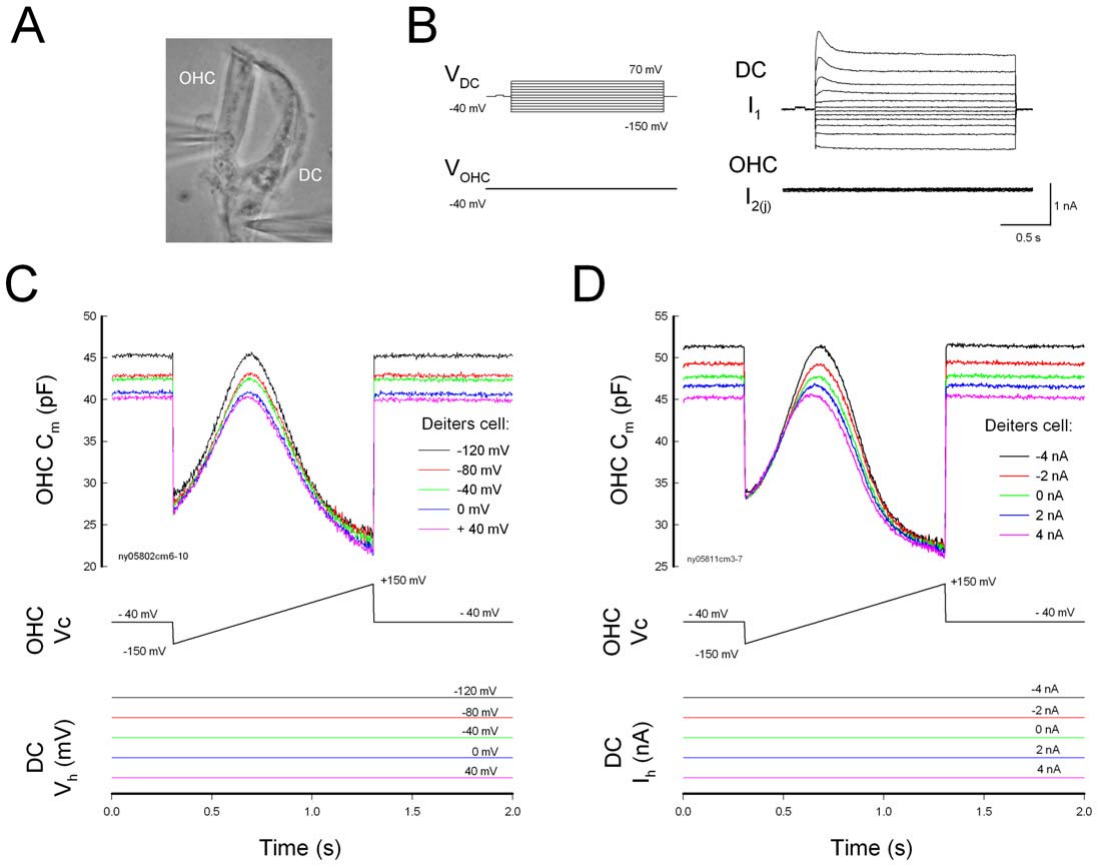
Modulation of Deiters cell (DC) membrane potential and current on outer hair cell (OHC) electromotility. A: A micrograph of double patch clamp recording between the DC and OHC in a DC-OHC pair. B: Double patch clamp recording between the DC and the OHC. There is neither transjunctional current nor electric conductance between the DC and the OHC. C: Changes in the holding potential of the DC alter OHC electromotility associated nonlinear capacitance (NLC). Both DC and OHC were recorded under voltage clamp. The bottom traces represent the different holding potentials at the DC. D: Changes in the holding current of the DC alter OHC NLC. The Deiters cell was clamped at different currents under the current clamp.

Deiters cells are physically linked with OHCs (Fig. 1A). Double patch clamp recording shows that there is no direct electrical coupling between the DCs and the OHCs (Fig. 1B). When giving voltage stimulations in the DCs, there was no transjunctional current or conductance between the DCs and the OHCs (Fig. 1B, n = 34).

However, electric stimulations in DCs could affect OHC electromotility (Fig. 1C and D). Fig. 1C shows that changes in holding potential of DCs altered OHC electromotility associated electrical signature NLC. Depolarization of DCs reduced NLC. The OHC NLC and the slope (*α*) of voltage dependence were reduced by 8.38±2.82% and 13.5±3.69% (n = 6, p = 0.005, paired t-test), respectively, as DCs were depolarized from −40 mV to +40 mV, and increased by 6.19±1.6% and 8.3±1.9% (n = 6, p = 0.004, paired t-test), respectively, for DCs hyperpolarized to −120 mV. Current stimulations in DCs also altered the OHC NLC (Fig. 1D). Cationic currents, which depolarize cells, reduced the NLC and its voltage dependent slope. The NLC and the voltage slope (α) were reduced by 13.2±2.1% and 11.4±2.7% (n = 7, p = 0.009, paired t-test), respectively, for holding current of DCs changing from −4 nA to +4 nA. The V_pk_ also had a significant shift by −21.75±12.72% and 14.56±6.26% for −4 nA and +4 nA current stimulations, respectively.

**Figure 2 pone-0007923-g002:**
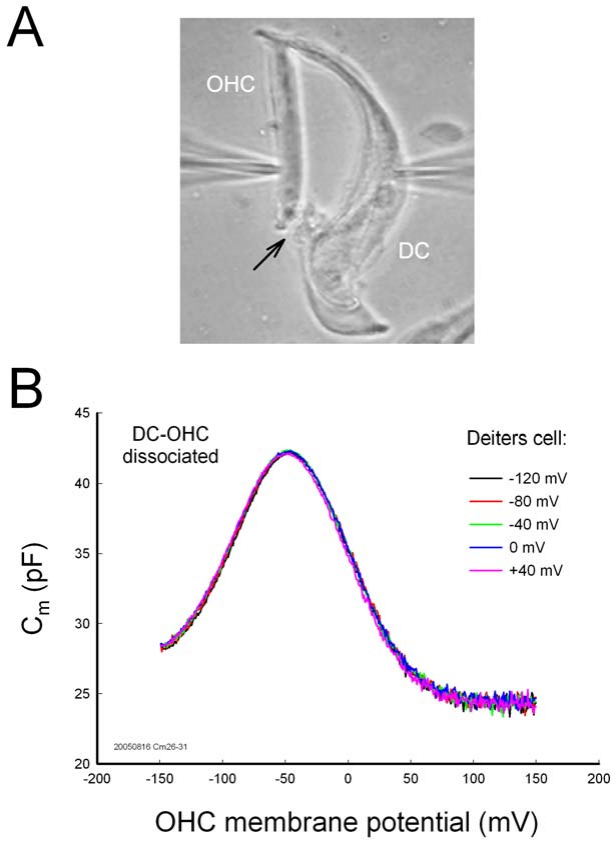
Breaking of mechanical connection between DC and OHC abolishes the effect of DC membrane potential on OHC electromotility. A: A micrograph of double patch clamp recording in a pair of DC-OHC. An arrow indicates dissociation of mechanical connection between the DC and the basal pole of the OHC. B: Dissociation abolishes the effect of DC membrane potential on OHC electromotility.

Disassociation of the mechanical-coupling between DCs and OHCs abolished this effect (Fig. 2). No change in NLC was observed in the dissociated DC-OHC pairs (Fig. 2B, n = 9), or after the DC-OHC physical connection was mechanically broken by the pipette (n = 4); the dissociation could be either at the apical connection between OHC cuticular plate and DC process or at the basal connection between OHC basal pole and DC body. This indicates the effect of electric stimulation in DCs on OHC electromotility through the DC-OHC mechanical coupling rather than through the effect of the extracellular electric field, which was clamped to zero (ground) in patch clamp recording.

**Figure 3 pone-0007923-g003:**
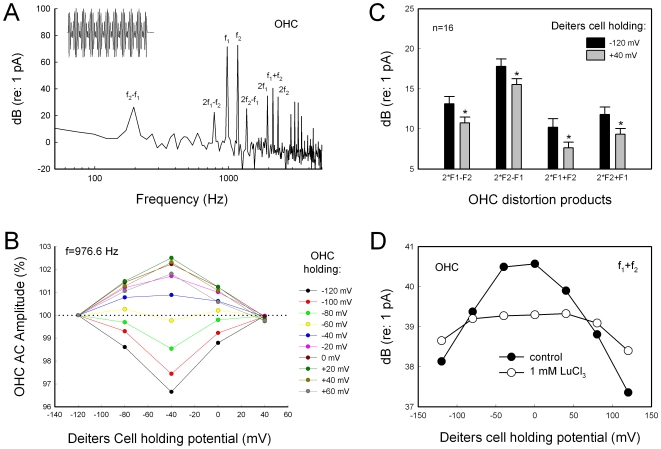
The effect of DC membrane potential on the OHC frequency responses and distortion products. A: The frequency spectrum and distortion products of OHC response to a two-sinusoidal electrical stimulation (f_1_ = 976.56 Hz, f_2_ = 1171.87 Hz, and V_p−p_ = 25 mV) in patch-clamp recording. Inset: A waveform of OHC response. B: The amplitudes of OHC frequency responses at different DC holding potentials. The amplitudes are normalized to those at DC holding potential of −120 mV. C: OHC distortion products at different holding potentials of the DCs. Depolarization of the DCs decreased OHC distortion products. Asterisks indicate p<0.05 (t test). D: Membrane potentials of DCs alter the OHC distortion product. The sum frequency distortion (f_1_+f_2_) was displayed. Perfusion of 1 mM LuCl_3_ reduced the effect.

Electric stimulation in DCs also altered the OHC frequency responses and distortion products (Fig. 3). Fig. 3A shows OHC frequency response and the generated distortion products measured by patch clamp recording. Depolarization or hyperpolarization of DCs altered OHC frequency responses (Fig. 3B). The OHC frequency response was also dependent upon OHC membrane potential because OHC electromotility is dependent upon membrane potential as well. Membrane potential of DCs also influenced OHC distortion products (Fig. 3C). Depolarization of DCs significantly reduced cubic distortion products by 15–25% (p<0.05, t test, n = 16). Fig. 3D shows that the effect of the DC membrane potential on a sum frequency distortion (f_1_+f_2_) of the OHC. Application of 1 mM LuCl_3_, which can inhibit OHC electromotility, reduced this effect.

### The effect of gap junctional coupling between DCs on OHC electromotility

**Figure 4 pone-0007923-g004:**
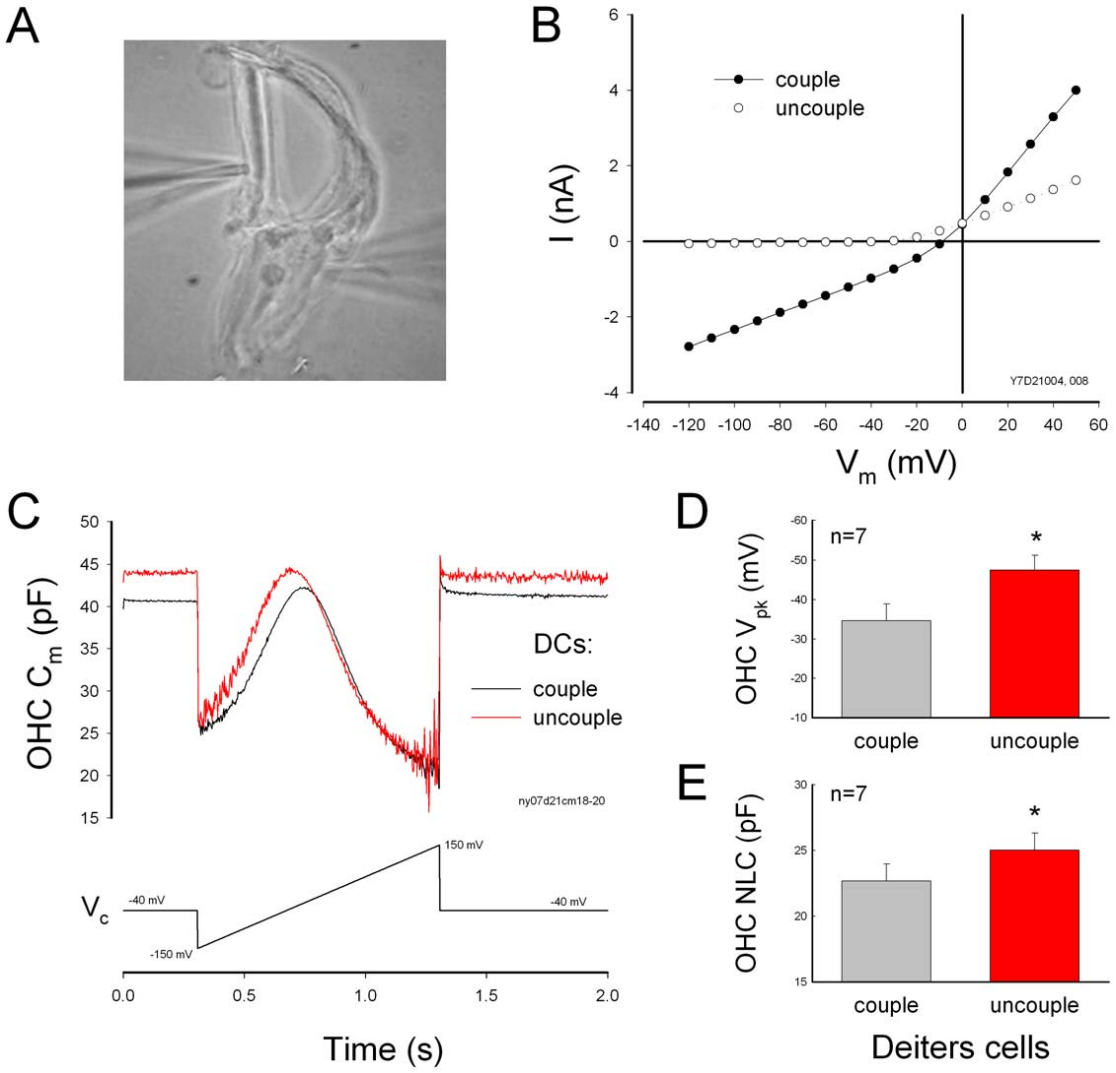
The effect of gap junctional coupling between DCs on OHC electromotility. A: A micrograph of a DC-OHC pair. An OHC is connected with two DCs. B: Membrane potential and current changes in DCs by uncoupling of gap junctions. Uncoupling reduced DC's membrane current and shifted zero-current potential to negative. C: Influence of gap junctional coupling between DCs on OHC NLC. The black and red lines represent the OHC membrane capacitance measured at DCs coupling and uncoupling, respectively, which was achieved by mechanical breaking. Uncoupling of gap junctions between DCs shifted the OHC NLC curve to negative. The V_pk_ was −21.8 and −40.3 mV and the NLC was 22.5 and 23.6 pF for DC coupling and uncoupling, respectively. D–E: Uncoupling of gap junctions between DCs induced changes in OHC NLC and V_pk_-shift.

There is no gap junctional coupling between DCs and OHCs (Fig. 1B). However, DCs are well-coupled by gap junctions [3]–[4], [16]. Uncoupling of gap junctional coupling between DCs could induce large changes in the membrane potential and current of DCs; uncoupling reduced the membrane current and shifted the membrane potential to the hyperpolarization direction (Fig. 4B, also see ref. 16). The zero-current membrane potential was shifted from −21.4±1.54 mV (n = 17) at coupling to −49.2±3.45 mV (n = 10) after uncoupling by mechanical-breaking one cell with a patch pipette. As shown by electric stimulations in DCs (Fig. 1C and D), the uncoupling of gap junctions between DCs caused significant changes in OHC NLC (Fig. 4C–E). The V_pk_ of the NLC shifted from −34.6±4.25 mV to −47.2±3.77 mV (n = 7, p<0.001, paired t-test). NLC was also increased by 15.0±6.3% (Fig. 4E). However, as shown by Fig. 2, after dissociation of mechanical connection between DCs and OHCs, the uncoupling gap junctions between DCs did not influence OHC electromotility (data not shown).

### Destruction of DC cytoskeleton abolishes the effect of DCs on OHC electromotility

**Figure 5:**
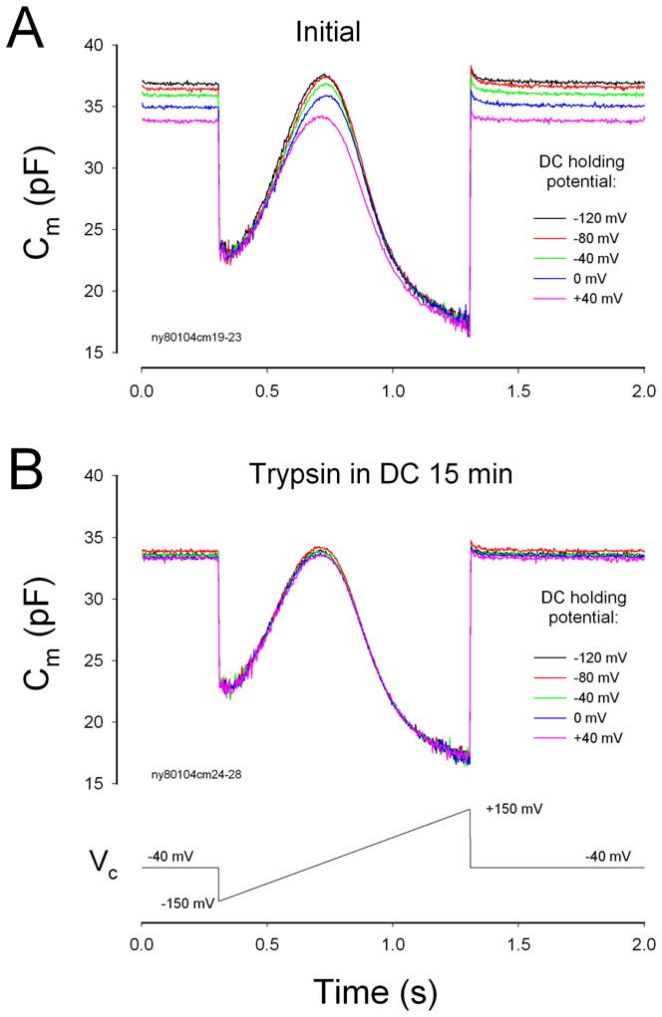
Destruction of DC cytoskeleton eliminates the effect of electrical stimulations in the DCs on OHC electromotility. Trypsin (0.25%) was only filled into the DC patch pipette. A: The DC holding potential can alter NLC at the beginning of whole-cell recording. B: The OHC NLC was recorded at 15 min after formation of whole-cell recording. The effect of DC membrane potential on NLC disappeared.

DCs contain a tubulovesicular membrane system and plentiful mitochondria, and can alter the phalangeal curvature by electric stimulus and ATP [17]–[19]. Destruction of DC cytoskeleton eliminated the effect of electric stimulations in DCs on OHC electromotility. Fig. 5 shows that the patch pipette in the DC was filled with 0.25% trypsin. Voltage stimulation in the DCs could alter NLC at the beginning of recording (Fig. 5A). After 15 min, the effect was abolished. The same voltage stimulation could not alter OHC NLC (Fig. 5B, n = 11). This implies that the cytoskeleton in DCs plays a critical role in the modulation of OHC electromotility by DCs. This also provides further evidence that the electric stimulations in DCs influence OHC electromotility through the DC-OHC mechanical coupling rather than by the extracellular electric field potential.

### The effect of mechanical stimulations in DCs on OHC electromotility

**Figure 6:**
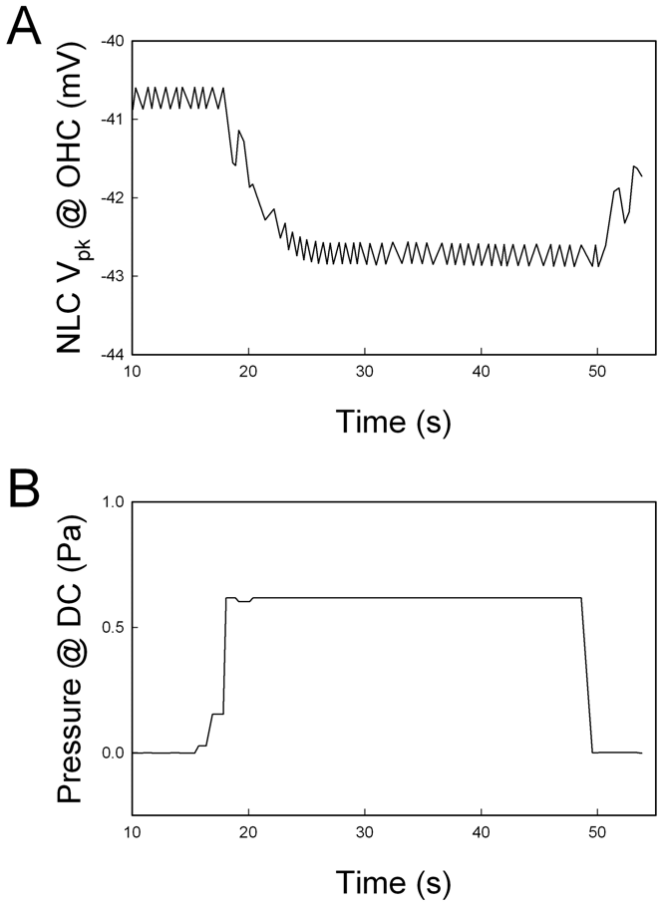
The effect of turgor pressure changes in the DC on OHC electromotility in a DC-OHC pair. The peak voltage (V_pk_) of NLC in the OHC was continuously recorded by use of a phase-tacking technique (Panel A). The DC was voltage-clamped at −40 mV under the whole-cell configuration and its turgor pressure was altered through the patch pipette (Panel B). Increase of turgor pressure in the DC caused the V_pk_ of OHC NLC shifted to negative voltage.

Mechanical stimulation in the DCs can also affect OHC electromotility. Fig. 6 shows that change in DC turgor pressure in the mechanical-coupled DC-OHC pairs could alter OHC electromotility. Alteration of turgor pressure can induce DC stalk process movement. Increase in the turgor pressure in the DCs reduces the curvature of the phalangeal stalk (i.e., the stalk elongates). Fig. 6A shows that the V_pk_ of NLC in the OHC was shifted to the negative direction when the turgor pressure in the DCs was increased through the patch pipette (Fig. 6B).

## Discussion

Cochlear supporting cells have been reported to play an important role in the control of hearing sensitivity in vivo [1], [2]. However, the cellular mechanism underlying this modulation remains unclear. Mammalian hearing relies upon active cochlear mechanics to amplify acoustic stimulations increasing hearing sensitivity and frequency selectivity. Two mechanisms have been proposed about active cochlear mechanics [8], [20]. One is the prestin-based OHC soma electromotility [21] and another is the stereocilium-based hair bundle movement. In this study, we found that DCs can modulate OHC NLC and frequency responses via mechanical coupling (Figs. 1–5). These data reveal a new cellular mechanism in the regulation of OHC electromotility.

1It has been reported that the DCs have microfilaments along the stalk process. They appear to be of opposite polarity, facilitating motility [5]. The DCs also contain a tubulovesicular membrane system and have plentiful mitochondria, implying a requirement for high levels of energy consumption and activity. Indeed, elevation of the intracellular concentration of Ca^++^ in DCs can trigger the stalk process movement and increases its stiffness [17]. In vivo, the stalk process of the DCs acts as a bow supporting OHC standing on DC cup (Fig. 1A). Such movement and changes can consequently alter OHC loading and membrane tension and modify OHC electromotility, because OHC has piezoelectricity [22], [23] and OHC electromotility has membrane tension dependence [9]–[11].

In this study, we found that depolarization of DCs reduced OHC NLC and shifted V_pk_ to the positive direction (Fig. 1C and D). Electrical stimulation can alter the curvature of the phalangeal stalk of the DCs [18], [19]. Depolarization induces the phalangeal stalk contracted to increase the process curvature [19]. This can compress OHCs to increase membrane tension and shifts the voltage dependence of OHC electromotility to the positive direction [9]–[10], [22]. We also found that increase in the turgor pressure in DCs shifted the V_pk_ of NLC to the negative direction (Fig. 6). Increase of turgor pressure in the DCs can elongate its process and reduces the curvature. As a result, this may reduce the compression in the OHC and decrease membrane tension to shift the V_pk_ of NLC negatively (Fig. 6). This also indicates that mechanical stimulation in the DCs can directly affect OHC electromotility via the mechanical-coupling.

This concept is further supported by the fact that dissociation of DC-OHC mechanical-coupling or destruction of the DC cytoskeleton abolished this influence (Figs. 2 and 5). These data also imply that the influence of electric stimulation in DCs on OHC electromotility is not mediated by the extracellular electric field, which has been grounded (clamped to zero) under whole-cell configuration in the patch clamp recording. Actually, we found that the patch pipette in the bath solution near the OHC could not evoke the detectable changes in OHC electromotility and NLC (data not shown). We have also demonstrated that a small transient (<1 ms) electric cross-talk at on-off stages (due to a small coupling capacitance generated by two closed patch pipettes) cannot influence OHC electromotility [23]. In the experiments, we used an ionic blocking solution which blocked OHC ionic channel activity. So, the possible local ionic changes in the DC cup area near the OHC basal pole cannot evoke the significant electric changes in OHCs. Thus, DCs directly affect OHC electromotility via the mechanical coupling between DCs and OHCs. This affection can eventually influence hearing sensitivity in the inner ear [1], [2].

In vivo, DCs are well-coupled by gap junctions [3], [4], which can synchronize and enhance this modulation on a large scale and play an important role in the control of hearing sensitivity [1], [2]. Change in gap junctional coupling between DCs also greatly alters the membrane potential and current in the DCs and influences OHC electromotility (Fig. 4; also see ref. [16]). Moreover, the cochlear supporting cells can release ATP through gap junctional hemichannels and regulate OHC electromotility by activation of purinergic P2x receptors [12], [13]. Recently, we have found that ATP can also mediate K^+^-sink in the cochlear supporting cells to recycle K^+^, which can produce a large inward current (up to several nano-amperes) in the supporting cells, including DCs, at the resting membrane potential [24]. Such current and voltage changes in the DCs can consequently regulate OHC electromotility via DC-OHC mechanical-coupling (Figs. 1–3). These new data further suggest that DCs can directly modulate OHC electromotility under the normal physiological conditions.

## References

[1] Fridberger A, Flock A, Ulfendahl M, Flock B (1998). Acoustic overstimulation increases outer hair cell Ca^2+^ concentrations and causes dynamic contractions of the hearing organ.. Proc Natl Acad Sci USA.

[2] Flock A, Flock B, Fridberger A, Scarfone E, Ulfendahl M (1999). Supporting cells contribute to control of hearing sensitivity.. J Neurosci.

[3] Forge A, Becker D, Casalotti S, Edwards J, Marziano N (2003). Gap junctions in the inner ear: comparison of distribution patterns in different vertebrates and assessement of connexin composition in mammals.. J Comp Neurol.

[4] Zhao HB, Yu N (2006). Distinct and gradient distributions of connexin26 and connexin30 in the cochlear sensory epithelium of guinea pigs.. J Comp Neurol.

[5] Slepecky NB, Dallos P, Popper AN, Fay RR (1996). in *The cochlea*,.

[6] Brownell WE, Bader CR, Bertrand D, Ribaupierre Y (1985). Evoked mechanical responses of isolated cochlear outer hair cells.. Science.

[7] Ashmore J (2008). Cochlear outer hair cell motility.. Physiol Rev.

[8] Dallos P (2008). Cochlear amplification, outer hair cells and prestin.. Curr Opin Neurobiol.

[9] Iwasa KH (1993). Effect of stress on the membrane capacitance of the auditory outer hair cell.. Biophys J.

[10] Kakehata S, Santos-Sacchi J (1995). Membrane tension directly shifts voltage dependence of outer hair cell motility and associated gating charge.. Biophys J.

[11] He DZ, Dallos P (1999). Somatic stiffness of cochlear outer hair cells is voltage-dependent.. Proc Natl Acad Sci USA.

[12] Zhao HB, Yu N, Fleming CR (2005). Gap junctional hemichannel-mediated ATP release and hearing controls in the inner ear.. Proc Natl Acad Sci USA.

[13] Yu N, Zhao HB (2008). ATP activates P2x receptors and requires extracellular Ca^2+^ participation to modify outer hair cell nonlinear capacitance.. Pflugers Arch.

[14] Zhao HB, Santos-Sacchi J (2000). Voltage gating of gap junctions in cochlear supporting cells: evidence for nonhomotypic channels.. J Memb Biol.

[15] Santos-Sacchi J, Kakehata S, Takahashi S (1998). Effects of membrane potential on the voltage dependence of motility-related charge in outer hair cells of the guinea-pig.. J Physiol.

[16] Zhao HB (2000). Directional rectification of gap junctional voltage gating between Deiters cells in the inner ear of guinea pig.. Neurosci Lett.

[17] Dulon D, Blanchet C, Laffon E (1994). Photo-released intracellular Ca^2+^ evokes reversible mechanical responses in supporting cells of the guinea-pig organ of Corti.. Biochem Biophys Res Commun.

[18] Bobbin RP (2001). ATP-induced movement of the stalks of isolated cochlear Deiters' cells.. Neuroreport.

[19] Bobbin PP, Campbell J, Lousteau S, Mandhare M, Berlin CI, Hood LJ, Ricci A, (2002). Electrical and ATP induced movements of the phalangeal processes of isolated cochlear Deiters' cells.. Hair cell micromechanics and otoacoustic emissions.

[20] Hudspeth AJ (2008). Making an effort to listen: mechanical amplification in the ear.. Neuron.

[21] Zheng J, Shen W, He DZ, Long KB, Madison LD (2000). Prestin is the motor protein of cochlear outer hair cells.. Nature.

[22] Gale JE, Ashmore JF (1994). Charge displacement induced by rapid stretch in the basolateral membrane of the guinea-pig outer hair cell.. Proc Biol Sci.

[23] Zhao HB, Santos-Sacchi J (1999). Auditory collusion and a coupled couple of outer hair cells.. Nature.

[24] Zhu Y, Zhao HB (2009). ATP-mediated potassium recycling in the cochlear supporting cells.. Purinergic Signal (in press).

